# A Common Infection in a Highly Atypical Patient: Hematochezia From a Cytomegalovirus Colonic Ulcer in a Young and Healthy Immunocompetent Patient

**DOI:** 10.7759/cureus.44274

**Published:** 2023-08-28

**Authors:** Nouf Turki, Jacob Newman, Leen Raddaoui, Taylor Brewer, Bedoor Alabbas, Sarah Turki, Mamoun Younes, Marie L Borum, Samuel A Schueler

**Affiliations:** 1 Gastroenterology and Hepatology, George Washington University School of Medicine and Health Sciences, Washington, DC, USA; 2 Gastroenterology and Hepatology, King Abdulaziz University Hospital, Jeddah, SAU; 3 Internal Medicine, George Washington University School of Medicine and Health Sciences, Washington, DC, USA; 4 Division of Gastroenterology and Liver Diseases, George Washington University School of Medicine and Health Sciences, Washington, DC, USA; 5 Internal Medicine, George Washington University, Washington, DC, USA; 6 Gastroenterology and Hepatology, Sinai Hospital of Baltimore, Baltimore, USA; 7 Pathology, George Washington University School of Medicine and Health Sciences, Washington, DC, USA

**Keywords:** cytomegalovirus (cmv), cytomegalovirus colitis, immunocompetent adult, hematochezia, colon ulcer

## Abstract

Gastrointestinal (GI) cytomegalovirus (CMV) infections are far more common in immunocompromised as opposed to immunocompetent patients. Immunocompetent patients who develop GI tract CMV infections are typically older with medical comorbidities. As such, descriptions of GI CMV infections in younger immunocompetent patients are lacking. Here, we present a case of a GI CMV infection in a young and healthy immunocompetent patient.

A 41-year-old male with hyperlipidemia and hypothyroidism presented with painless, intermittent hematochezia. He denied changes in bowel habits or appetite, abdominal pain, fevers, chills, fatigue, or weight loss. His history was pertinent for insertive and receptive intercourse with one male partner. Medications were emtricitabine/tenofovir for pre-exposure prophylaxis, levothyroxine, and atorvastatin. A colonoscopy revealed a cecal ulcer surrounded by nodular-appearing mucosa that felt firm and friable when biopsied. The remaining colon and terminal ileum were normal. There was no diverticulosis or hemorrhoids. Pathology was positive for CMV. A subsequent serological evaluation revealed a normal complete blood count and comprehensive metabolic panel. Tests for human immunodeficiency virus, syphilis, viral hepatitis, chlamydia, and gonorrhea were negative. He was treated with valganciclovir 900 mg twice daily for 21 days. A subsequent test for CMV deoxyribonucleic acid polymerase chain was negative. Hematochezia resolved. A repeat colonoscopy revealed normal mucosa in the cecum.

GI CMV infections in immunocompetent patients are rare and typically occur in older patients with medical comorbidities. Further, such case reports are needed to inform clinicians about risk factors and the presentation of GI CMV infections in young healthy immunocompetent patients.

## Introduction

Cytomegalovirus (CMV) infections of the gastrointestinal (GI) tract are far more common in immunocompromised as opposed to immunocompetent patients. Immunocompetent patients who develop CMV GI tract infections are typically older with medical comorbidities. As such, descriptions of CMV GI infections in younger immunocompetent patients are lacking. Here, we present a 41-year-old immunocompetent male discovered to have a CMV-positive ulcer in the cecum.

This article was previously presented as a meeting abstract at the American College of Gastroenterology Conference on October 23, 2022.

## Case presentation

**Figure 1:**
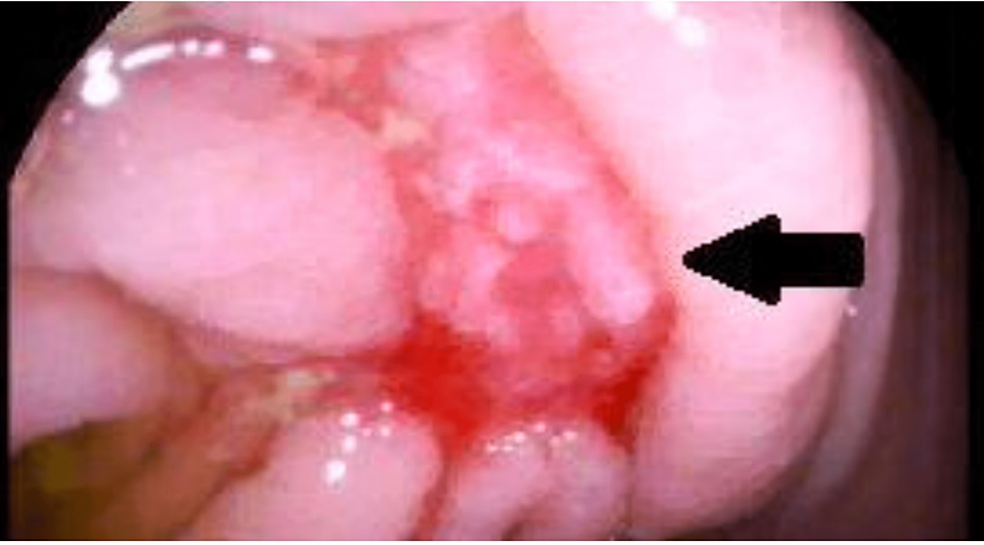
Ulcer with surrounding nodular mucosa (thick black arrow) to the right of the appendiceal orifice in the cecum.

**Figure 2:**
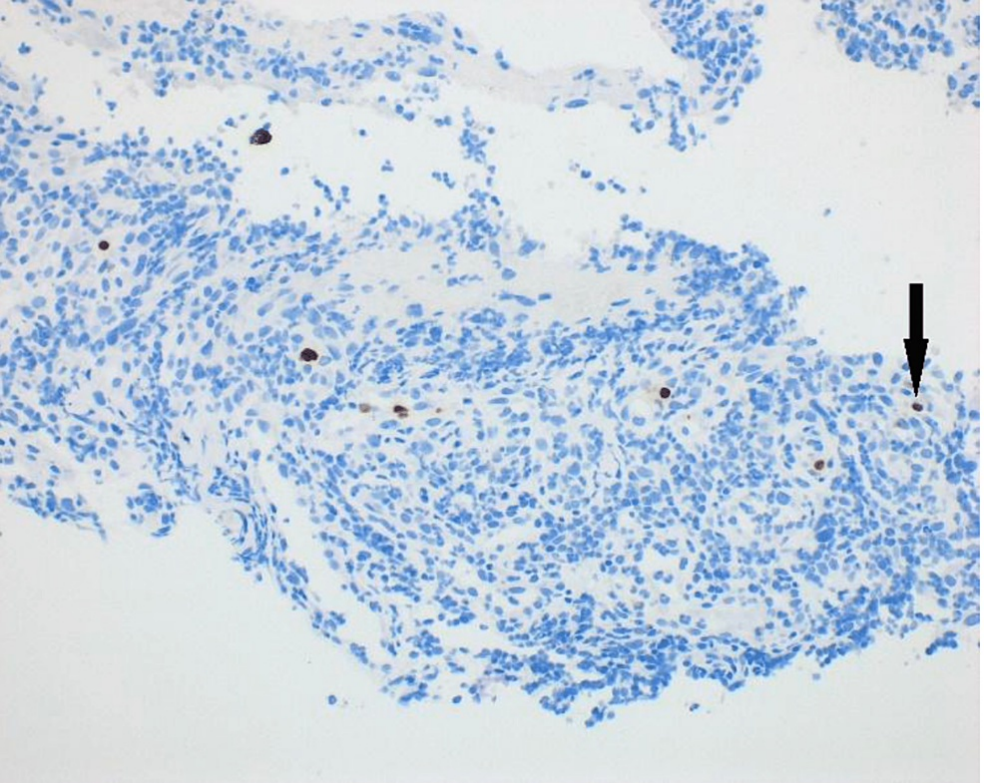
Immunohistochemical stain for cytomegalovirus (CMV) at 20× magnification. The brown nuclear stains correspond to CMV-positive cells (thin black arrow).

**Figure 3:**
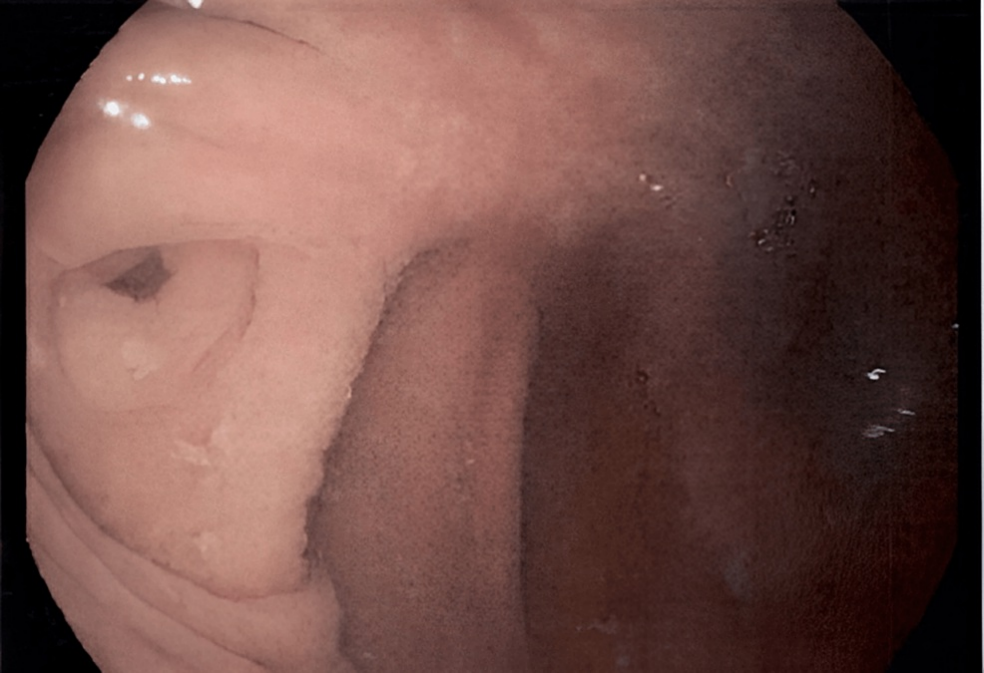
Healed cytomegalovirus ulcer post-treatment.

A 41-year-old male with hyperlipidemia and hypothyroidism presented to our outpatient department with painless, intermittent hematochezia. He denied changes in bowel habits, changes in appetite, abdominal pain, fevers, chills, fatigue, or weight loss. His social history was pertinent for insertive and receptive intercourse with one male partner and negative for smoking history. Medications included emtricitabine/tenofovir for men who have sex with men, for which he took emtricitabine/tenofovir for pre-exposure prophylaxis, levothyroxine, and atorvastatin. His family history included a maternal uncle with colon cancer diagnosed at age 60. His vital signs were normal, and his abdominal examination was benign. A colonoscopy revealed an area of nodular-appearing and ulcerated mucosa in the cecum that felt firm and friable when biopsied (Figure 1). The remaining colon, including the terminal ileum and rectum, were normal. There was no diverticulosis or hemorrhoids. Biopsies revealed ulcerations with immunoperoxidase staining positive for CMV (Figure 2). Subsequent serological evaluation revealed a normal complete blood count and comprehensive metabolic panel. Tests for human immunodeficiency virus, syphilis, viral hepatitis, chlamydia, and gonorrhea were negative. He was treated with valganciclovir 900 mg twice daily for 21 days. A subsequent test for CMV deoxyribonucleic acid polymerase chain was negative. Hematochezia resolved. A repeat colonoscopy revealed normal mucosa in the cecum (Figure 3).

## Discussion

In various reports analyzing the literature regarding immunocompetent individuals with CMV, it is evident that, on average, immunodeficient patients are much younger than their immunocompetent counterparts. One study used a MEDLINE search from 1966 through 1980 to identify 13 immunocompetent CMV patients, in addition to their own two case reports. They concluded the mean age to be 57.3 years old ranging from 22 to 85 years old (53.3% of whom were 67 years or older). Their analysis further described the mean age of patients with probable reactivation to be older (43.9 years old) compared to the mean age of patients with probable primary infection (43.9 years old) [1]. In another study, researchers compared 56 immunocompetent patients to 117 immunosuppressed patients and found the median age difference was 24.4 years, with the average age of immunocompetent patients being 73 years old and immunodeficient patients being 48.6 years old [2]. Additionally, a meta-analysis of CMV colitis in immunocompetent hosts published by Digestive Diseases and Sciences examined 44 cases and concluded the overall average age to be 61.1 years old ranging from 22 to 92 years old [3]. Although our case subject was relatively young compared to these averages, there are still many case reports that review patients in their 20s [4].

Regarding the ulceration pattern and comorbidities of the 41-year-old man presented in this case report, the patient’s isolated ulcer and comorbidities are not representative of the existing literature. This draws particular interest to this report as it may add to a greater literature review. Multiple erosive and superficial ulcerations were found to be more common than a singular pattern: for erosive, 73.3% compared to 13.3%, and for superficial, 84.6% compared to 15.4%, respectively [1]. In addition, the patient’s existing comorbidities of hypothyroidism and hyperlipidemia are not represented in the literature describing common comorbidities related to immunocompetent CMV patients. Renal failure, diabetes mellitus, large-vessel atherosclerosis, and non-hematological malignancies are commonly mentioned as comorbidities across the literature for immunocompetent CMV patients [1-3,5]. However, some studies have reported that chronic kidney disease stage and prevalence are not significantly different between immunocompetent and immunocompromised CMV patients [1]. Still, there are some studies that describe other comorbidities that may exist, such as pregnancy [5]. It is widely concluded that immunocompetent patients with described comorbidities are more likely to contract CMV than those without [1]. It is also shown that those without comorbidities are often younger and have a greater chance of spontaneous recovery and survival, suggesting that perhaps the comorbidities are the cause of older immunocompetent individuals becoming infected with CMV [2]. Lastly, upon seeking help, bloody stools and an infectious mononucleosis-like syndrome are common complaints among CMV immunocompetent patients [1,3].

## Conclusions

While CMV colitis is commonly associated with older individuals who have various medical comorbidities in the immunocompetent population, it is important to note that this condition can also manifest in younger individuals who are otherwise in good health. The process of diagnosing this condition might pose greater challenges in such cases, and the approach to treatment will hinge on the severity of symptoms and the presence of any underlying medical conditions.
